# Cloning and expression of a novel α-galactosidase from *Lactobacillus amylolyticus* L6 with hydrolytic and transgalactosyl properties

**DOI:** 10.1371/journal.pone.0235687

**Published:** 2020-07-17

**Authors:** Yongtao Fei, WenJuan Jiao, Ying Wang, Jinglong Liang, Gongliang Liu, Li Li

**Affiliations:** 1 College of Light Industry and Food Science, Zhongkai University of Agriculture and Engineering, Guangzhou, China; 2 Guangdong Academy of Agricultural Sciences, Sericultural & Agri-Food Research Institute, Guangzhou, China; 3 School of Food Science and Engineering, South China University of Technology, Guangzhou, China; Technical Educational Institute of Peloponnese, GREECE

## Abstract

*Lactobacillus amylolyticus* L6, a gram-positive amylolytic bacterium isolated from naturally fermented tofu whey (NFTW), was able to hydrolyze raffinose and stachyose for the production of *α*-galactosidase. The cell-free extract of *L*. *amylolyticus* L6 was found to exhibit glycosyltransferase activity to synthesize α-galacto-oligosaccharides (GOS) with melibiose as substrate. The coding genes of *α*-galactosidase were identified in the genome of *L*. *amylolyticus* L6. The α-galactosidase (AglB) was placed into GH36 family by amino acid sequence alignments with other α-galactosidases from lactobacilli. The optimal reaction conditions of pH and temperature for AglB were pH 6.0 and 37°C, respectively. Besides, potassium ion was found to improve the activity of AglB while divalent mercury ion, copper ion and zinc ion displayed different degrees of inhibition effect. Under the optimum reaction condition, AglB could catalyze the synthesis of GOS with degree of polymerization (DP) ≥5 by using 300 mM melibiose concentration as substrate. The maximum yield of GOS with (DP) ≥3 could reach 31.56% (w/w). Transgalactosyl properties made AglB a potential candidate for application in the production of GOS.

## Introduction

α-galactosidase (EC 3.2.1.22), also named melibiase, is able to hydrolyze α-1,6 linkage between glucose and galactose in raffinose and stachyose [1]. There are two kinds of hydrolytic mechanism for α-galactosidase, including retaining and inverting mechanism. Many α-galactosidases belonging to families 4, 27, 36 and 57 present a retaining mechanism of hydrolysis, which were found to have transglycosylated abilities under high substrate concentration [2]. The similar phenomenon was also found in the β-galactosidases with high concentration of lactose as substrate [3, 4].

Currently, α-galacto-oligosaccharides (GOS) have been identified as prebiotic ingredients for their numerous health benefits by large amount of vitro and vivo studies in animal and human body [5]. Using α-galactosidase to synthesize GOS was widely reported and considered as one of the most effective ways. Two novel α-galactosidase from *Mesorhizobium* and *Streptomyces* exhibited transglycosylation activity, which required different acceptors to produce different GOS [6]. Furthermore, thermophilic α-galactosidase from *Talaromyces leycettanus* JCM12802 was successfully expressed in *Pichia pastoris* GS115 and found with transglycosylation capacity, owning great potentials in the synthesis of GOS [7]. Previous studies found that *α*-galactosidase existed in most of lactic acid bacteria, whereas *α*-galactosidases with glycosyl transferase activity were mostly reported in Bifidobacteria [8–11]. A new *α*-GOS molecule (*α*-D-Galp-(1–6)-*α*-D-Galp-(1–6)-D-Glcp) that could promote the growth of probiotics in intestine was synthesized by α-galactosidases from *Bifidobacterium adolescentis* [8]. Besides, Goulas et al. found that the whole cells of *Bifidobacterium bifidum* NCIMB 41171 could be used to catalyze the synthesis of α-GOS and β-GOS by *α*-and *β*-galactosidase [12]. The gene coding for α-galactosidase from *Bifidobacterium bifidum* NCIMB 41171 was cloned and expressed, and the recombinant enzyme was found with transgalactosylating properties [10]. Most of the reported α-galactosidase catalyzed the synthesized reaction with Gal-α-1,6 linkage, whereas Zhao et al. showed that Aga2 from *Bifidobacterium breve* 203 was able to synthesize globotriose analogues with Gal-α-1,4 linkage, acting as high-affinity inhibitors for Shiga toxin [11]. However, *α*-galactosidase with transglycosylation activity from lactobacilli was rarely reported. The recently reported *α*-galactosidase with transglycosylation activity was from *Lactobacillus plantarum* WCFS1 [13].

The starting point of this research is *L*. *amylolyticus* L6, isolated from naturally fermented tofu whey (NFTW) in which the main carbon sources were raffinose and stachyose [14]. This strain owned strong ability of hydrolyzing raffinose and stachyose thanks to the production of *α*-galactosidase [15]. Beside the hydrolytic activity, we found that the crude enzyme of *α*-galactosidase exhibited partial transglycosylation activity. The complete genome of *L*. *amylolyticus* L6 has been sequenced, and the genes responsible for *α*-galactosidase (AglB) were identified [16]. Therefore, the objectives of this research were to clone and express putative genes coding for α-galactosides, characterize the enzymatic properties, then investigate its transglycosylation activity to explore its potential in the production of GOS.

## Materials and methods

### Chemicals and bacterial strains

Plasmid isolation kit, Taq Polymerase, PrimeSTAR^®^ HS DNA Polymerase, dNTP and isopropyl-1-thio-b-galactopyranoside (IPTG) were purchased from Takara (Dalian, China). Substrate p-nitrophenyl α-D-galactopyranoside (pNPG) was obtained from Sigma Chemical Co. (Saint Louis, MO, USA). HisTrap HP was purchased from GE Healthcare Bio-Sciences (Connecticut, Fairfield, USA). The culture media including (de Man,Rogosa,Sharpe) MRS and Luria Bertani (LB) were purchased from Guangdong Huankai Microbiology Biotech Inc. (Guangzhou, China). All other chemicals and reagents were analytical grade. MRS (de Man, Rogosa, Sharpe) and LB (Luria Bertani) broth (g/L) were prepared according to our previous report.

The strain *L*. *amylolyticus* L6 isolated from NFTW has been shown to be a potential probiotic with application in fermenting tofu whey [15, 16]. *L*. *amylolyticus* L6 was preserved in 15% (v/v) glycerol at -80°C and activated in 10 mL MRS broth at 37°C for 36 h. *Escherichia coli* DH5α and BL21 (DE3) were used for recombinant plasmid construction and gene expression, respectively. Both *E*. *coli* strains were cultured in LB agar or broth at 37°C under aerobic conditions.

### Preparation of α-galactosidase crude extract and its assays

The activated *L*. *amylolyticus* L6 was incubated in modified MRS replacing glucose with equal amount of 1% raffinose to induce the production of α-galactosidase. The procedures of preparing α-galactosidase crude extract were carried out according to our previous reports [15, 16]. Briefly, cells of *L*. *amylolyticus* L6 were collected by centrifugation (8000 g×10 min) at room temperature, and washed two times with citrate phosphate buffers (pH 5.5), then collected at the same condition of centrifugation. The cell pellets were resuspended and treated with 6 mg/mL lysozyme at 37°C for 1 h in water bath. The treated cells were harvested by centrifugation (8000 g×10 min) at 4°C and resuspended in pre-cooling citrate phosphate buffer. The cell-free extracts were obtained by disrupting the cell suspensions with ultrasonic cell crusher (55 w, 2 s/2 s, 10 min). The supernatant containing α-galactosidase was collected by centrifugation (8000 g×15 min) at 4°C. The α-galactosidase assay was carried out with pNPG as substrate and citrate phosphate buffers (pH 5.5) as reaction buffer [15]. The reaction mix was incubated at 37°C for 30 min and stopped with Na_2_CO_3_. Microplate spectrophotometer was used to determine the absorbance of reaction mix at 405 nm. One unit of activity was defined as the amount of enzyme that release 1.0 μmol pNP from pNPG per minute.

### Construction of recombinant plasmid

DNA of *L*. *amylolyticus* L6 was obtained with DNA extraction kit and used as template of PCR. The Expand High Fidelity PCR System with PrimeSTAR^®^ HS DNA Polymerase (Takara, Dalian, China) was used to clone gene *aga1* (Accession number: CP020457) with oligonucleotides: 5’- CCG GAATTCATGAA CCACGAACTAATCAC-3′ containing a *EcoR* I site (underlined) and 5′-CCG CTCGAGTTAATTCCGTACACTGTTTG-3′ containing a *Xho* I site (underlined). The DNA fragmentof *aglB* was amplied and then purified with PCR Purification Kit (Axygen, Guangzhou, China). The resulting products were sent to Takara Biotechnology (Takara, Dalian, China) for sequencing. After that, the purified DNA products were digested by *EcoR* I and *Xho* I, and cloned into expression vector pET-32a. The recombinant plasmid pET-gal was transformed and propagated in *E*. *coli* DH5a.

### Expression and purification of α-galactosidase

The recombinant plasmid pET-gal was transformed into *E*. *coli* BL21 (DE3) and cultured at 37°C in LB medium with 50 μg/mL ampicillin. When OD_600_ of the culture reached 0.5 in logarithmic phase, 1mM of IPTG was added in the medium and cultured at 30°C, 180 rpm for 4 h to induce the expression of AglB. Cells were collected by centrifugation at 4°C, 8000 g for10 min, then cleaned and resuspended in pre-cooling McIlvaine (pH 5.8). The resulting cells were disrupted by ultrasonication (55 w, 2 s/2 s, 10 min) in ice bath and then centrifuged at 4°C, 8000 g for 10 min. The supernatant filtered was equilibrated and washed with 10 mM imidazole and 20 mM HEPES (pH 7.5), and eluted with imidazole concentration of 400 mM. The imidazole elutions detected with AglB activity were collected and precipitated by cold acetone (-20°C). The precipitation of protein was then resuspended in McIlvaine buffer. The above steps were carried out under 4°C. The spectrophotometric method was applied to determine the protein concentration at 280 nm. The molecular mass of expressed AglB was further determined by SDS-PAGE with stain of Coomassie Brilliant Blue.

### Characterization of enzymic properties for AglB

The effect of temperature, pH, and metal ions on AglB activity were performed according to previous descriptions [17, 18]. The optimal reaction pH of AglB with pNPαGal as substrate was carried out in 50 mM McIlvaine buffer with pH range of 3.0 to 8.0. 20 μl AglB was incubated in 500 μl pNPαGal (10 mM) in McIlvaine buffer at 37°C for 10 min. As for optimal reaction pH, the relative activity (100%) was the ratio of enzymic activity at different pH with the maximum enzymic activity at optimal pH. Meanwhile, the pH stability of AglB was determined with appropriate amount of AglB incubated at above pH range at 15°C for 2 h. After that, pNPαGal was added into the reaction mix and incubated for 10 min to detect the activity of AglB. In respects of pH stability, the relative activity (100%) was the ratio of enzymic activity after 2 h incubation at different pH with the enzymic activity before 2 h incubation at different pH.

The optimal temperature and thermal stability of AglB were further determined. The optimal temperature was performed in McIlvaine buffer (pH 5.0) with 20 μl enzyme, 500 μl of 10 mM pNPαGal in the temperature range from 20°C to 45°C for 10 min. 0.5 mL of sodium carbonate (1.0 M) was added to stop the reaction, and the enzymic activity was then measured at 37°C under the wave length of 405 nm. Thermal stability of the enzyme was detected by incubating AglB at temperature range from 20°C to 45°C for 2 h. During the incubation, 20 μl aliquots were taken from reaction mix at 20 min intervals, and the enzymic activity was measured with the same method.

The purified AglB was incubated in the reaction mix supplemented with metal ions such as Na^+^, K^+^, Ca^2+^, Cu^2+^, Fe^2+^, Mn^2+^, Mg^2+^, Zn^2+^ at 37°C for 10min. The concentration of metal ions in the reaction mix was 10mM. Besides, the effect of EDTA on activity of AglB was also tested with concentration gradient of 10 mM in the McIlvaine buffer (pH 5.0). The enzyme activity without addition of metal ion was used as control. All experiments were carried out in triplicate.

### Transgalactosylase activity and analysis of GOS content

Transgalactosylase activity of AglB was carried out with 300 mM melibiose as substrate in McIlvaine buffer at pH 5.0. The reaction mix were incubated at 37°C in a water-bath shaker with 150 rpm. The samples were taken at 3 h, 6 h, 12 h, 18 h, and 24 h, and the enzyme activity in the samples was immediately inactivated by heating at 95°C for 10 min. The resulting samples were analyzed by high-performance liquid chromatography (HPLC) using HP-NH_2_ column (4.6×250 mm, American) and refractive index detector. The parameters were as follows: the mobile phase 68%—acetonitrile32% water, flow rate 1.0 mL/min, temperature 36°C, sample size 20 μL and separating time 26 min. The concentration of monosaccharides and disaccharides were determined using standard calibration curves of galactose, glucose and melibiose. The degree of polymerization (DP) of GOS were further analyzed by liquid chromatograph mass spectrometer (LC-MS) with Waters ACQUITY UPLC® BEH HP-NH_2_ column (2.1×100mm, 1.7μm). The parameters were as follows: electrospray ionization (ESI) with anion model, solvent removal vapor temperature of 180°C, sample taper hole voltage 20 V, collision energy 6 eV, cone gas flow 8L/min, extraction cone hole voltage 3 V, Capillary voltage 3500 V, and the detection range of molecular weight 100–1500 Da.

### Statistical analysis

Data are expressed as the mean standard deviation (SD) of three replicates. Significant differences between the means of parameters were calculated with Duncan’s multiple-range test using SPSS 17.0 software (SPSS, Inc., Chicago, IL, USA). p< 0.05 was considered statistically significant.

## Results and discussion

### Sequence analysis of α-galactosidase genes from *L*. *amylolyticus* L6

**Fig 1:**
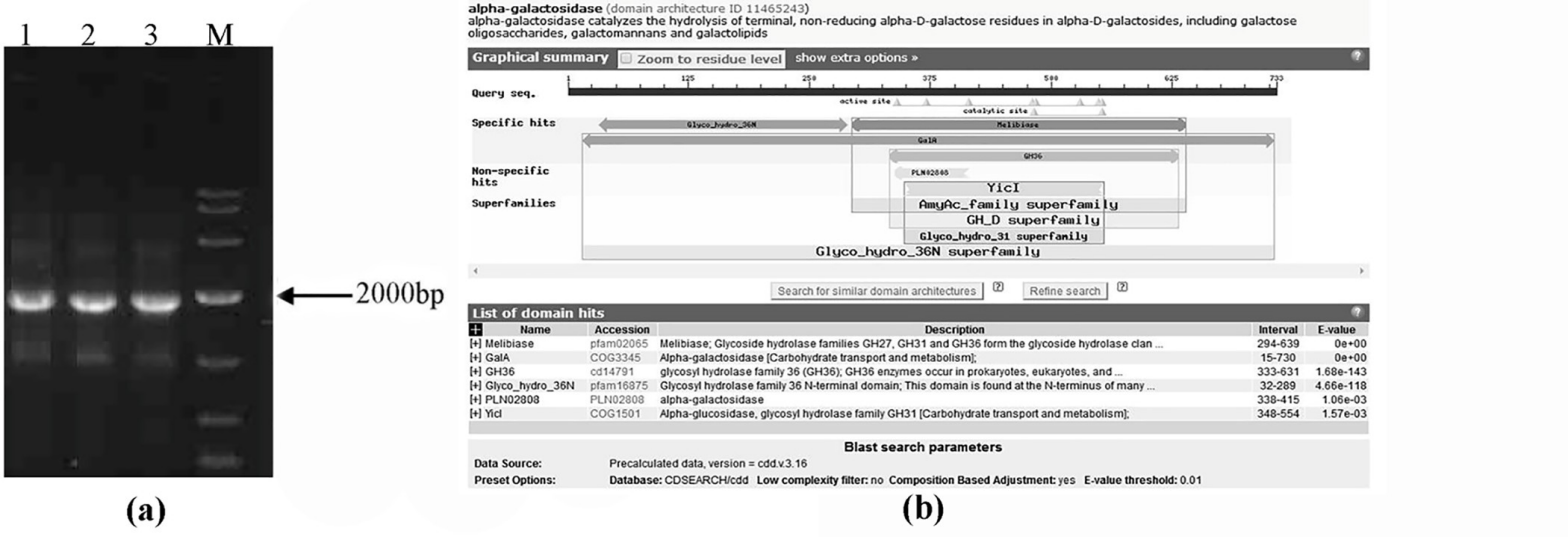
Gel electrophoresis of PCR products of *α-gal* gene. (a) (lane 1–3 represent PCR products of *α-gal* gene, M represents DNA Marker DL2000) and amino acid sequences analysis of AglB by BLASTn.

The complete genome of *L*. *amylolytcus* L6 has been sequenced and reported in our previous research [16]. Based on the annotation of genome sequence, there are four genes coding α-galactosidase (*α-gal*), including L6_1618 (2202 bp), L6_1632 (399 bp), L6_1633(1815 bp) and L6_1645 (2202 bp). Interestingly, all the *α-gal* genes were not found in the chromosome but located in plasmid pBL118, indicating that *α-gal* genes of *L*. *amylolytcus* L6 were obtained by horizontal gene transfer from other bacteria. The acquisition of *α-gal* genes could help *L*. *amylolytcus* L6 adapt the environment of tofu whey where raffinose and stachyose were the main carbon source. Because of the instability of plasmid in the host strain, the *α-gal* genes were easy to be lost during the propagation without selective pressure of raffinose and stachyose, leading to instability of α-galactosidase expression in *L*. *amylolytcus* L6. Expression of *α-gal* gene in *E*. *coli* BL21 can obtain large amounts of purified α-galactosidase for the study of enzymic properties, which are more efficient and time-saving. Further analyzing DNA and amino acid sequences of α-galactosidase in the BLAST (https://blast.ncbi.nlm.nih.gov/Blast.cgi) indicated that L6_1618 and L6_1645 (2202 bp) were two copies of *α-gal* genes while L6_1632 (399 bp) and L6_1633(1815 bp) only have partial sequence of *α-gal* genes. Besides, the amino acid sequence of AglB that deduced from *α-gal* (L6_1618) displayed high similarity with that of α-galactosidase from *Lactobacillus hamsteri* (83%), *Lactobacillus amylovorus* (79%), *Lactobacillus johnsonii* (79%). Based on the amino acid sequence identity, α-galactosidase can be classified into six enzyme families, including GH4, GH27, GH36, GH57, GH97 and GH110 [19]. AglB of *L*. *amylolyticus* L6 was placed into family GH36 to which most of bacteria-derived α-galactosidase belonged (Fig 1B). It has been reported that most α-galactosidases of families 36 are retaining glycosidases mechanism, some of which are found to exhibit transglycosylation activity [2].

According to the DNA sequence of L6_1618, the primers GLA-F and GLA-R were designed and applied to amplify target DNA gene with High Fidelity PCR System. The PCR products were detected by agarose gel eletrophoresis, indicating there is a single band with the size of approximately 2000 bp in the gel (Fig 1A). The PCR product was purified and then sequenced, affirming that the sequence was in accordance with that of L6_1618.

### Expression and purification of AglB

**Fig 2:**
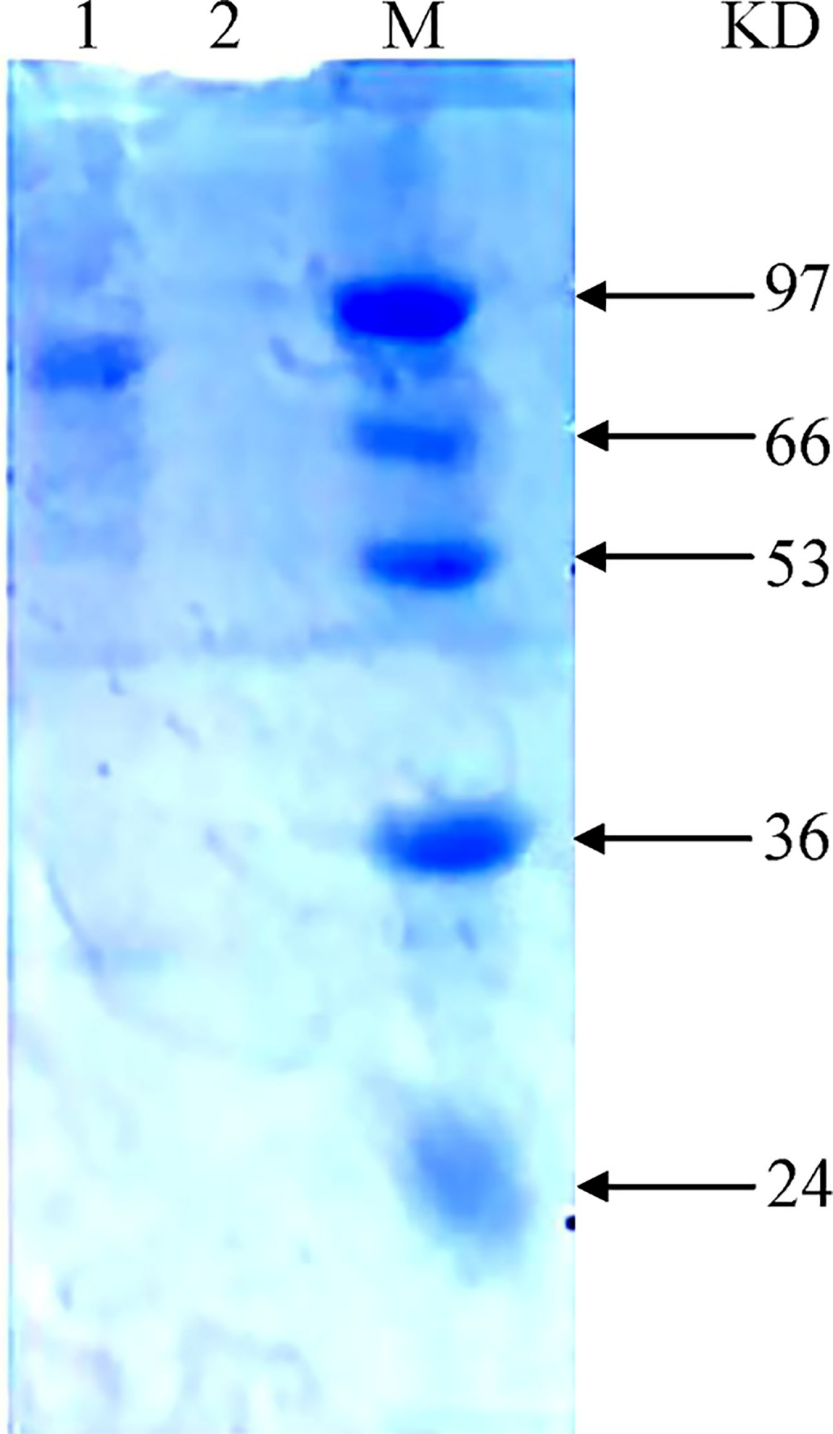
SDS-PAGE detection of heterologously expressed AglB purified with affinity chromatography. (M, Marker; lane 1, purified AglB protein; lane 2, control).

Unlike most of fungal GH36 *α*-galactosidases with signal peptide, promoting the excretion of enzyme out the cell [1, 20], our previous results indicated that AglB from *L*. *amylolytcus* L6 is an intracellular enzyme [14]. The gene fragment of AglB obtained by PCR was ligated into expression vector pET-32a, producing recombinant plasmid pET-gal that was further identified by double enzyme digestion (data not shown). The recombinant plasmid was transformed into *E*. *coli* BL21(DE3) and induced by IPTG. The resulting infused AglB with His-tag was purified by nickel chelating chromatography with yield of 500 mg from 1000 mL medium. The hydrolysis activity of AglB was detected with pNPG method, indicating that we have successfully expressed *aglB* gene in *E*. *coli* BL21(DE3). The purified AglB was determined with SDS-PAGE, showing a single band with estimated molecular mass of 80 kDa (Fig 2).

### Enzymatic properties of AglB

**Fig 3:**
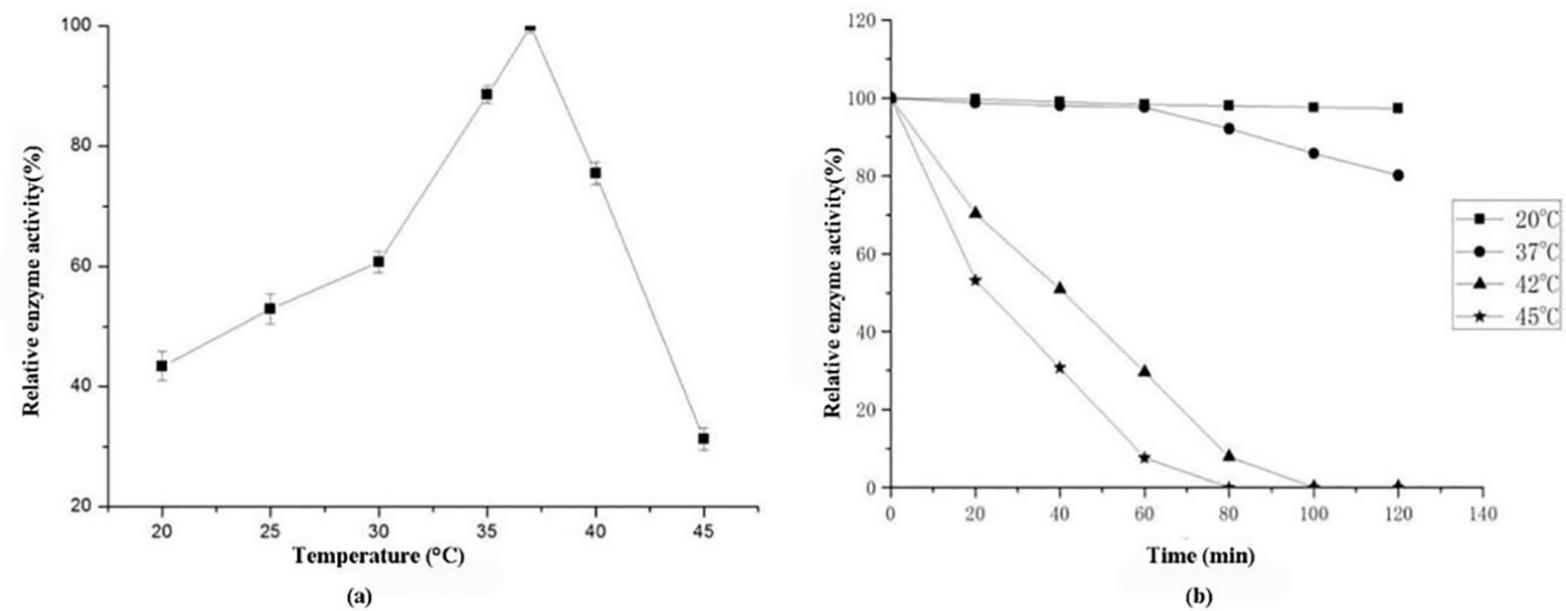
The optimum reaction temperature of AglB using pNPG as substrate (a) and its thermal stability (b).

The optimal temperature and thermal stability of AglB were investigated as shown in Fig 3. The results showed that the optimum temperature for AglB was 37°C (Fig 3A), which was different from optimum growth temperature (42°C) of *L*. *amylolyticus* L6 [15], further indicating that *aglB* gene in the plasmid of *L*. *amylolyticus* L6 was acquired from other symbiotic bacteria. The optimum temperature of AglB was similar to that of α-galactosidase from *Lactobacillus helveticus* and *Haliotis corrugate* [17, 21] but different from 55°C of *L*. *fermentum* [18] and 90°C of *S*. *solfataricus* [22]. Besides, AglB exhibited 100% stability at 20°C for 120 min (Fig 3B) while it lost 25% and 50% of activities at 42°C and 45°C respectively after only 20 min incubation. That might be due to the thermal denaturation of AglB protein under higher temperature. Therefore, AglB was not suitable in the application of the field with highly thermal stability requirement but it can be useful in vivo at 37°C in the form of oral capsule to eliminate flatulence caused by raffinose or stachyose and synthesize prebiotics like GOS simultaneously.

**Fig 4:**
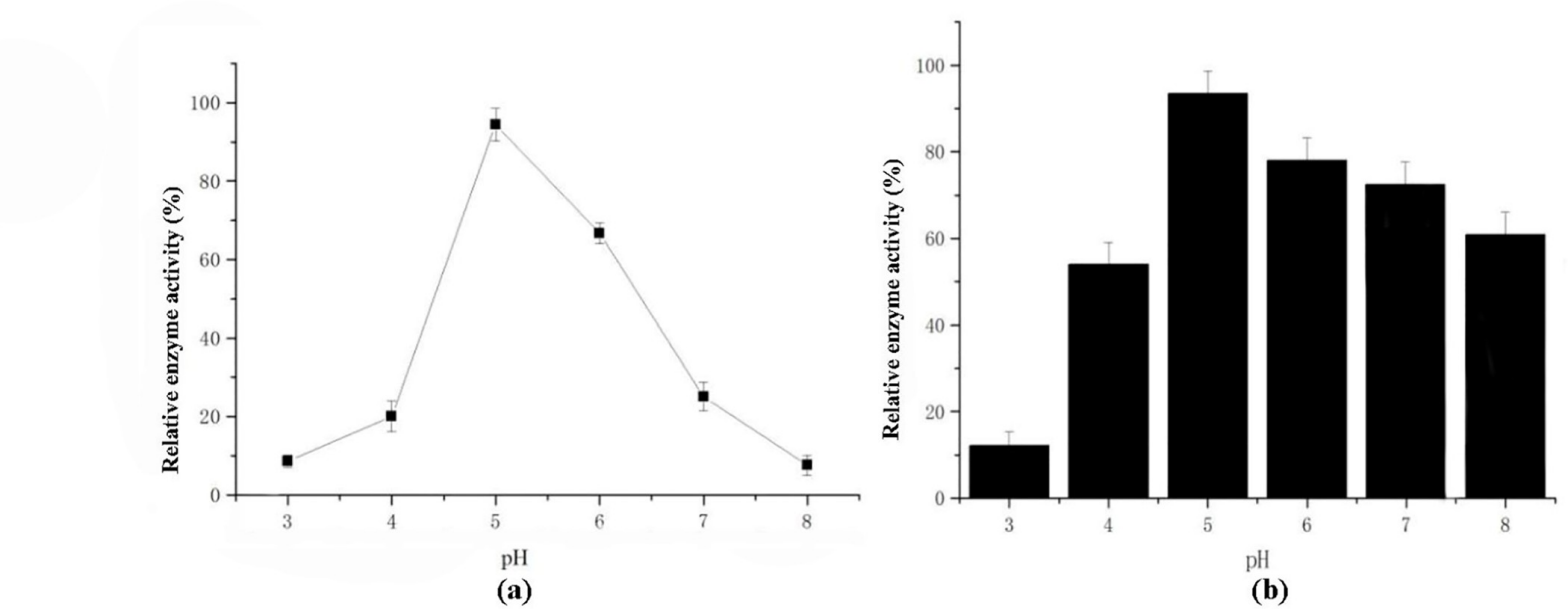
The optimum reaction pH of AglB (a) and its stability at different pH (b).

The optimum pH for AglB was 5.0 (Fig 4A), which was similar to that of *L*. *fermentum* CRL722 [18] and *Lactobacillus reuteri* [23]. But some α-galactosidase from lactobacilli, such as *L*. *helveticus* ATCC 10797 and *L*. *fermentum* CRL 251, exhibited maximum activity at pH 6.0 and lost activity below the pH of 4.0 [17]. Besides, this enzyme exhibited good stability between pH 5.0 and 8.0 after 2h incubation. But the stability of AglB reduced evidently at pH 3 with only 12% activity (Fig 4B). Therefore, the stability of this enzyme under neutral pH could reduce the side reactions of the monosaccharide in the enzyme processing technology.

As shown in Table 1, different metal cations including monovalent and divalent ions exhibited various effects on the activity of AglB. Of the tested cations, Hg^2+^ displayed strongest inhibition on the activity of AglB with only 35% activity remained. Besides, divalent ions, such as Cu^2+^ and Zn^2+^, also have an negative effect on the enzymatic activity. The inhibition of AglB might be due to the modification of sulfhydryl group by mercuric ion. Similar results have been reported that mercury exhibits evident inhibition on several α-Gal from *Lactobacillus fermentum* and *Lactobacillus helveticus* [17, 18]. Previous studies showed that Fe^2+^ was strong inhibitor for α-galactosidase from *Monascus pilosus* [24], but Fe^2+^ in this study has negligible effects on AglB. Other divalent ions such as Mg^2+^, Ca^2+^, and Mn^2+^, have no effect on activity of AglB, which was similar to that of α-galactosidase from *Lactobacillus fermentum* CRL722 [18]. However, several studies showed that Mn^2+^ and Ca^2+^ could inhibit the activity of α-galactosidase [25]. Besides, AglB in this study could also be activated by potassium ion (Table 1), and the similar result has been reported that potassium ion was able to improve the activity of α-galactosidase [17, 26].

**Table 1:** Effect of metal ions on AglB activity.

Metal ions	Relative enzyme activity (%)
H_2_O	100.0±2.9
Monovalent ions	
KCl	110.3±4.3
NaCl	99.1±1.3
LiCl	98.4±3.1
CsCl	96.8±5.0
Divalent ions	
CaCl_2_	93.4±1.6
CuSO_4_	62.6±2.6
MgSO_4_	92.4±6.8
MgCl_2_	96.2±4.1
MnCl_2_	97.4±1.6
MnSO_4_	94.4±2.2
FeSO_4_	97.1±2.5
ZnSO_4_	47.4±3.1
HgCl_2_	35.2±5.7

### Transgalactosylation properties of AglB

The crude extraction of α-galactosidase was used to explore the possibility of synthesizing GOS with melibiose as substrate. The result of HPLC indicated that there are two additional peaks after the peaks presenting glucose, galactose and melibiose, respectively. The retention time was in direct proportion with molecular weight of detected sugars. Besides, the area of two additional peaks gradually increased as the reaction time of crude enzyme prolonged. Therefore, the gene of AglB was heterologously expressed in *E*. *coli* BL21(DE3) to further investigate its transgalactosyl activity to synthesize GOS.

**Fig 5:**
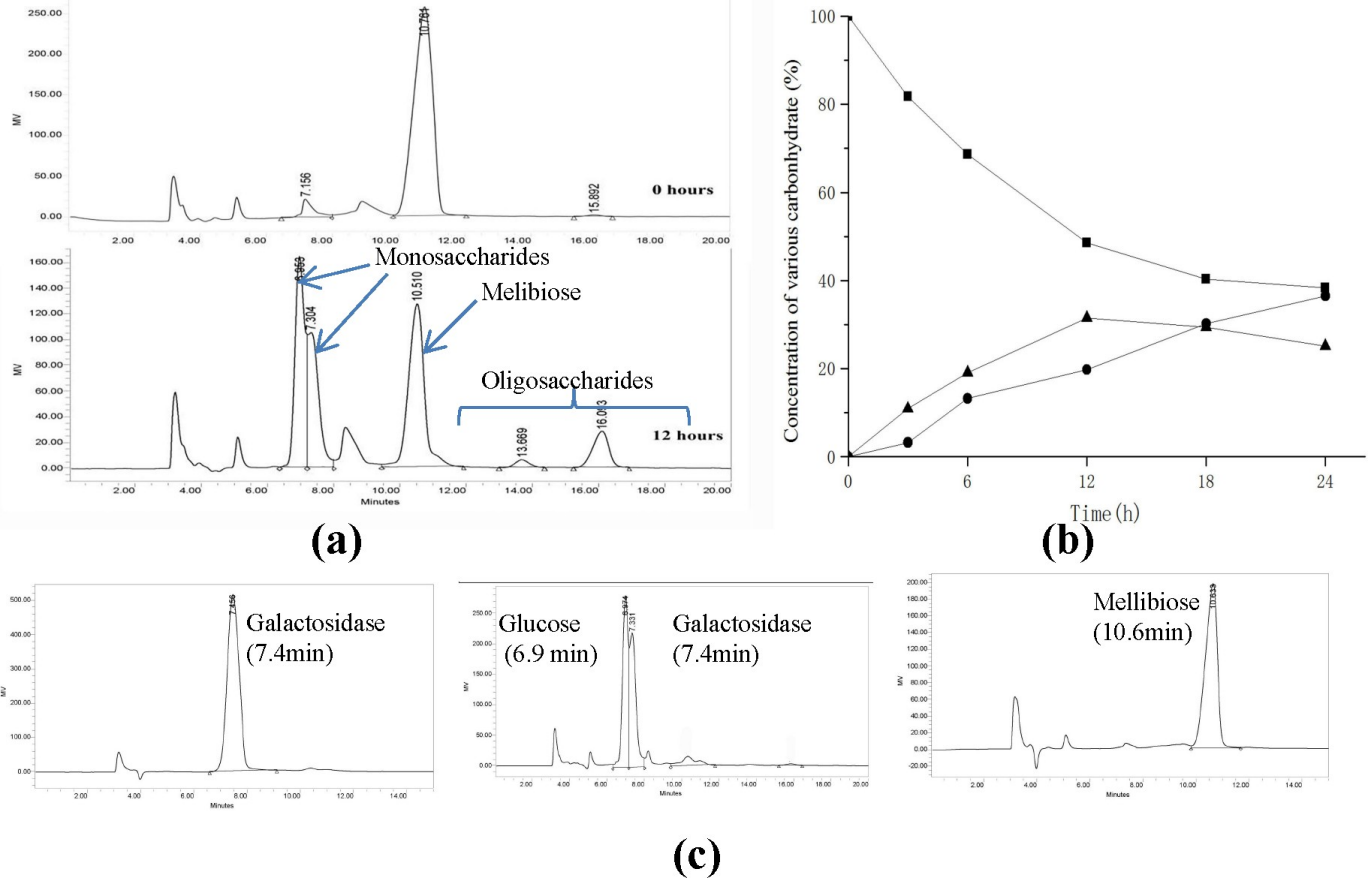
HPLC analysis of GOS synthesized by AglB (a), the time course of melibiose conversion (b), and the retention time of glucose, galactose, and melibiose (c). (■) Melibiose, (●) Monos accharide, (▲) Oligosaccharides (retention time13.7 min and 16.0 min).

**Fig 6:**
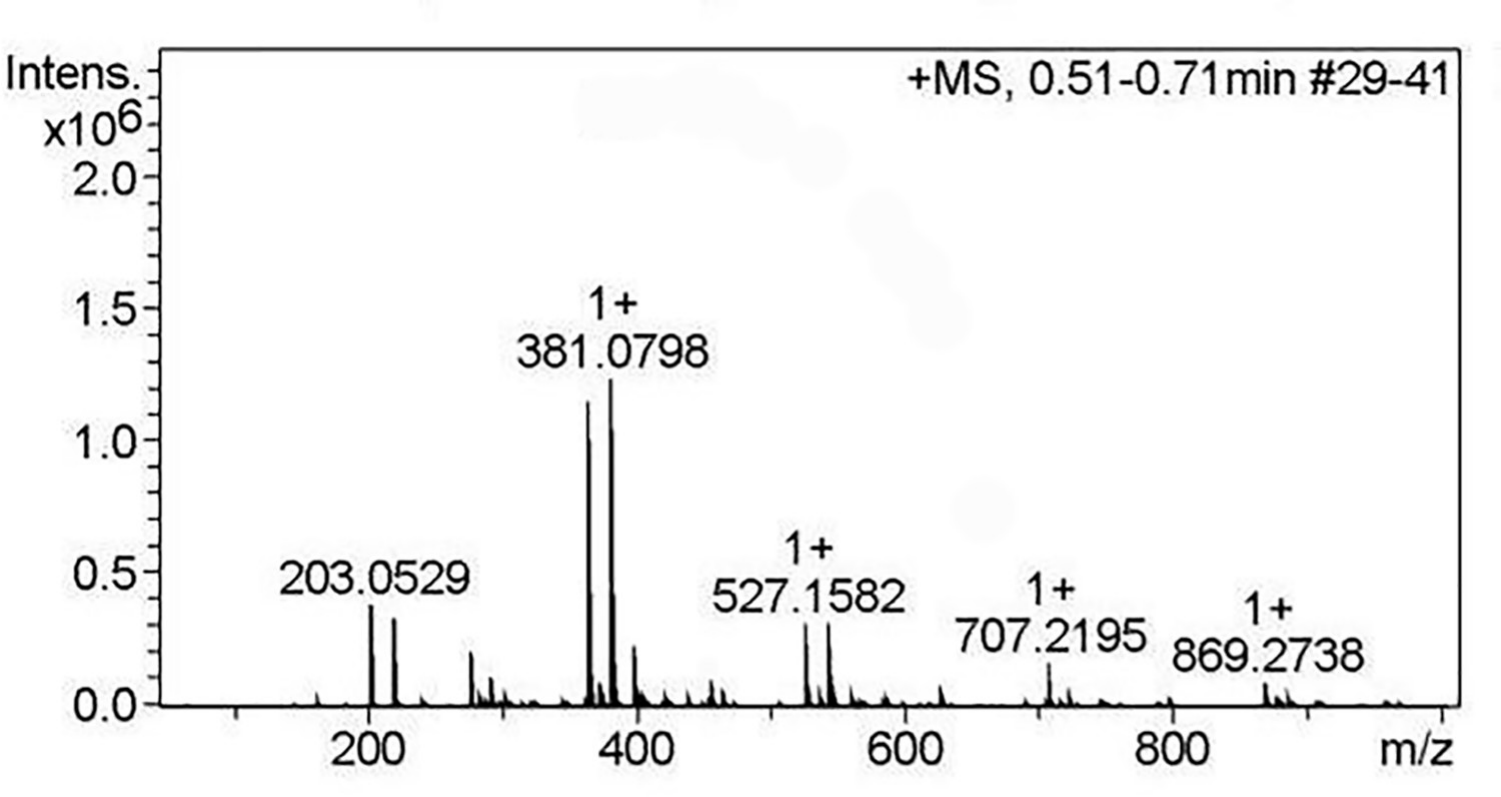
LC-MS spectra of the oligosaccharides synthesized by AglB with melibiose as substrate.

Transferase activity of the recombinant enzyme was performed at 37°C in reaction buffer with pH 5.0 which was optimum for activity and stability of AglB as shown in previous results. 300 mM of melibiose was used as the substrate of AglB according to previous research [10]. Compared with components in the reaction system at 0 h, the concentration of melibiose reduced with the production of monosaccharides (galactose and glucose) and oligosaccharides at 12 h (Fig 5A). Meanwhile, the conversion course of melibiose into monosaccharides and oligosaccharides was shown in Fig 5B. Following the transferase reaction of AglB, it was observed that the highest yield of oligosaccharides was obtained at 12 h. A maximum yield of oligosaccharide was 31.56% (w/w) with the melibiose conversion ration of 51.36% (w/w). Besides, AglB was favourable towards oligosaccharides with degree of polymerization (DP) ≥3 than that with DP = 2 as determined by HPLC. The yield of AglB was higher than that of α-galactosidase from *Βifidobacterium bifidum* (20.5%), *Lactobacillus helveticus* ATCC 10797 (26%) and *Bifidobacterium breve* 203 (10.7%) [10, 11, 17], but lower than that of *Bifidobacterium adolescentis* DSM 20083 (33%) [27]. LC-MS was used to determine DP of oligosaccharides (Fig 6). The result showed that m/z of 527 representing the oligosaccharides (DP = 3) in the form of sodium (M+23) was detected with the relative intensity of 25.3%. Meanwhile, m/z of 707 representing oligosaccharides (DP = 4) in the form of potassium ions (M+39) was detected with the relative intensity of 13.0%. Besides, oligosaccharides (DP = 5) in the form of potassium ions with m/z of 869 was also found in the products of AglB. Most of reported α-galactosidase was found to produce oligosaccharides with (DP) of 3 and 4 [20, 27], and α-galactosidase with the ability of synthesizing oligosaccharides with (DP) of 5 was rarely reported. AglB could not only synthesize oligosaccharides with (DP) of 3 and 4, but also be able to catalyze the production of oligosaccharides with (DP) of 5 with high concentration of melibiose as substrate.

## Conclusions

In this study, gene *aglB* coding for α-galactosidase was identified in the genome of *Lactobacillus amylolyticus* L6. Bioinformatic analysis showed that AglB belongs to GH36 family with the retaining hydrolytic mechanisms, thus owning the potential to catalyze reaction of transglycosylation. The gene *aglB* was cloned and successfully expressed in *E*. *coli* BL21 (DE3). The molecular mass of recombinant enzyme AglB was 328 kDa, and its optimal reaction temperature, pH and ion condition were also determined. Besides, AglB was found to have transglycosylation activity to synthesize GOS with 300 mM melibiose as substrate. The maximum yield of GOS with (DP) ≥3 was 31.56% (w/w) and the GOS with (DP) ≥5 was also detected in final products. The result of this study displayed that AgaB has the potential to be used in the production of GOS. However, the chemical bond of GOS synthesized by AglB should be investigated in the following research. Meanwhile, the optimal condition for the production of GOS should be performed with orthogonal experiment. Further, the prebiotic effect of galactooligosaccharides in promoting the growth of various intestinal bacteria, including various lactobacilli and Bifidobacteria.

## Supporting information

S1 File (PDF)
