# Supplementary figures and images for: Cloning and expression of a novel α-galactosidase from Lactobacillus amylolyticus L6 with hydrolytic and transgalactosyl properties
Source: PLoS One. 2020 Jul 17;15(7):e0235687. doi: 10.1371/journal.pone.0235687 (PMC7367483; doi:10.1371/journal.pone.0235687)

S1\_raw\_images

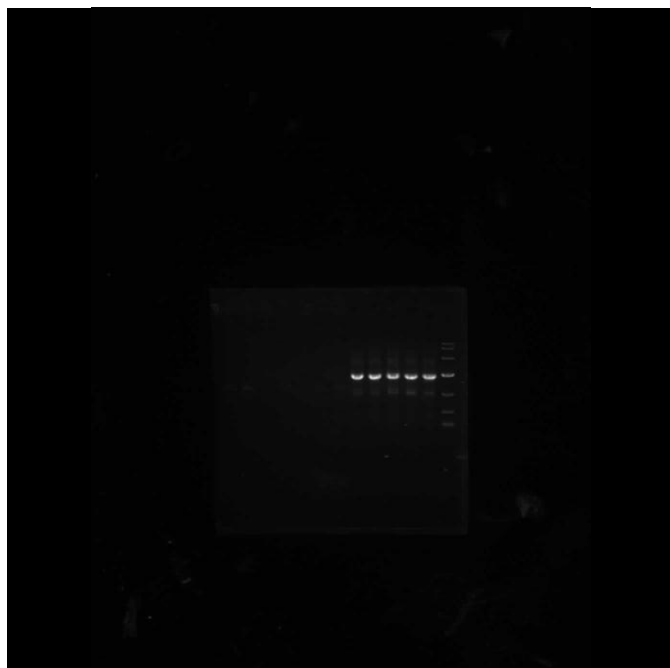

S2\_raw\_images

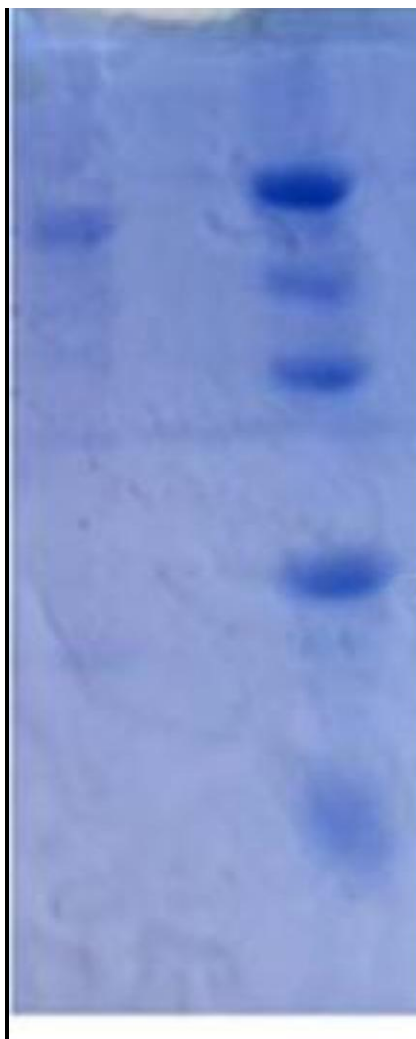

## S3\_raw\_images

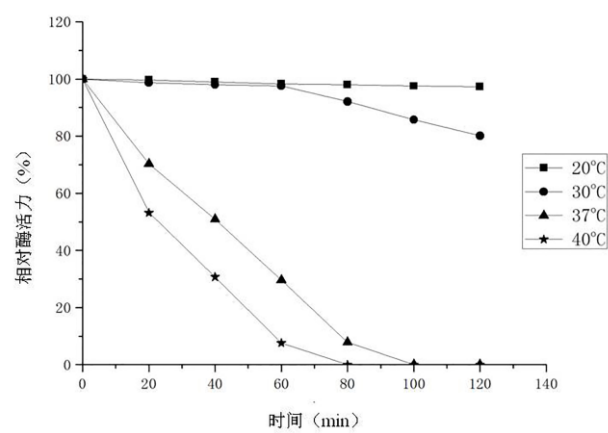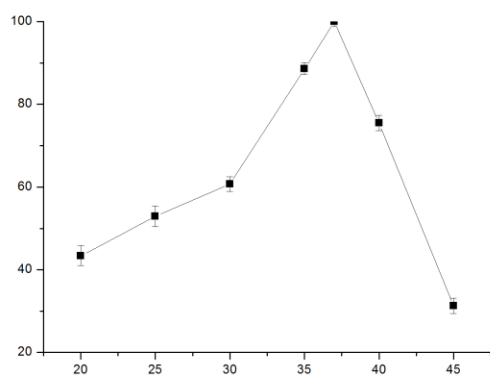

S4\_raw\_images

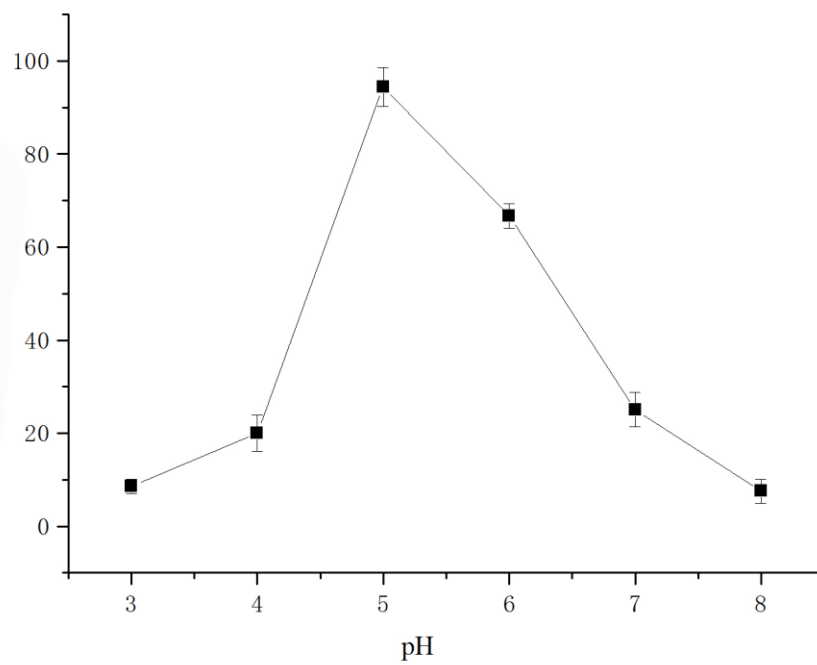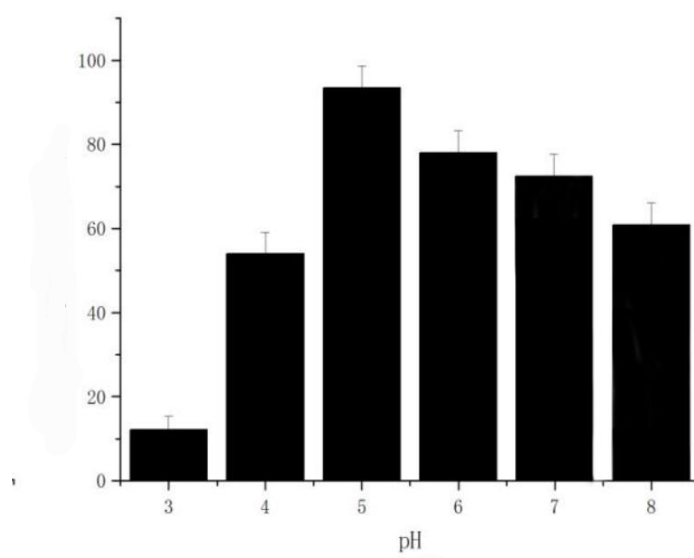

## S5\_raw\_images

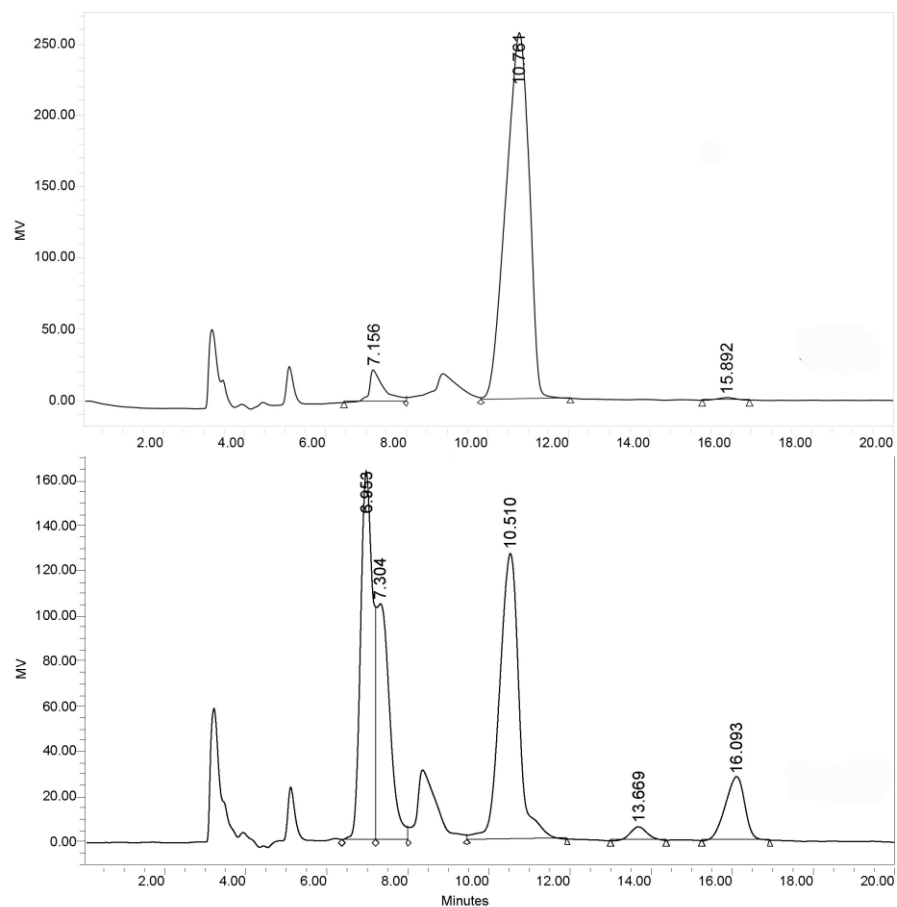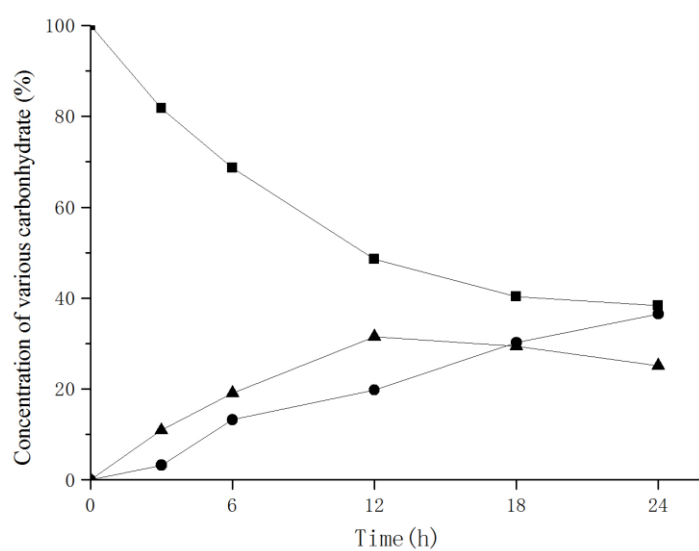

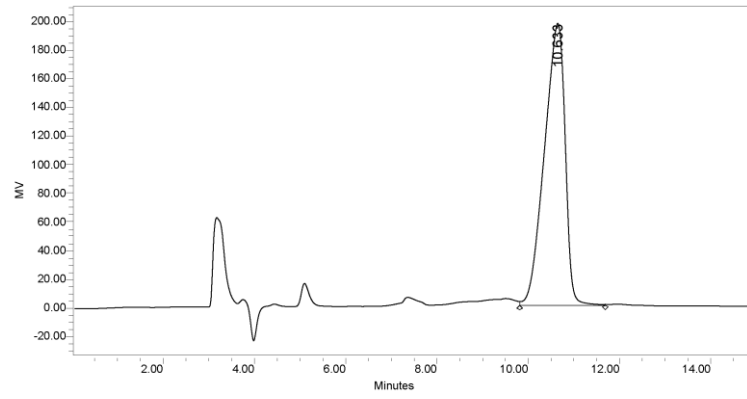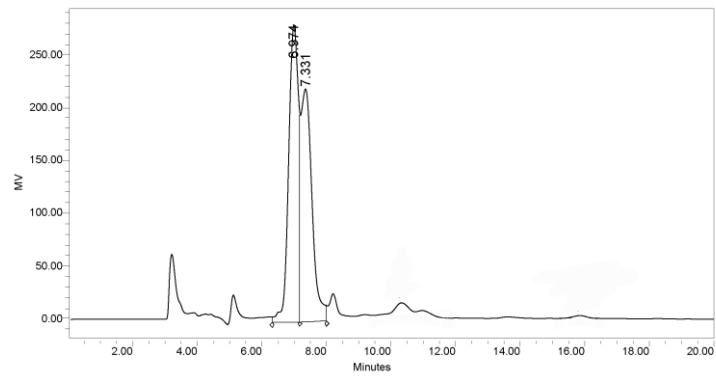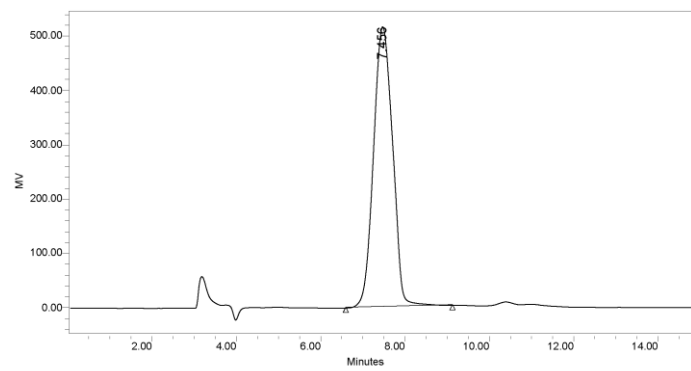

S6\_raw\_images

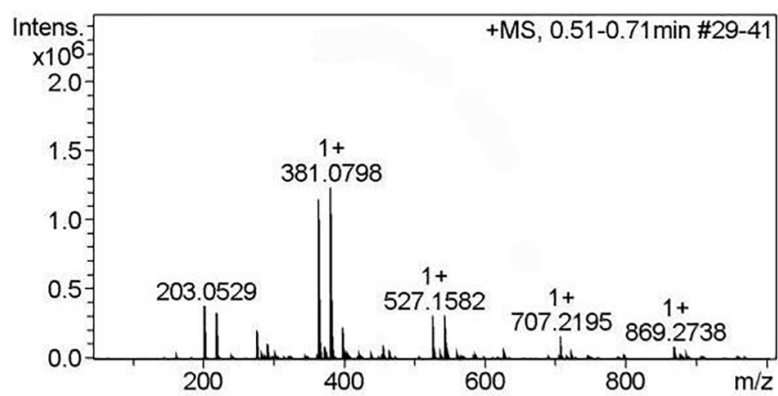

S S1\_raw\_images

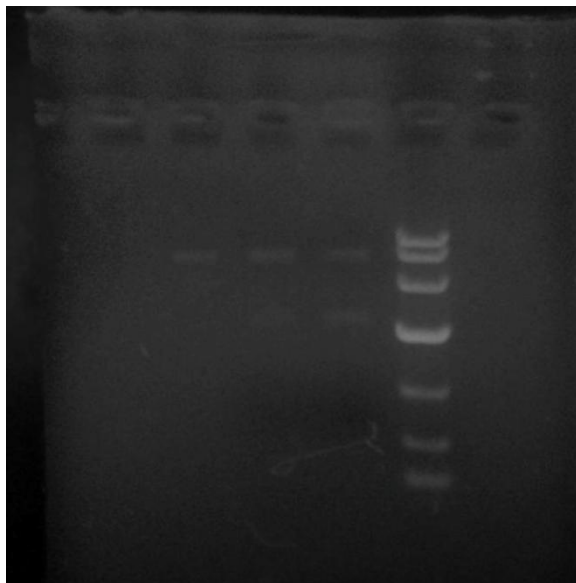

Supplement: S1 File — (PDF) [file pone.0235687.s001.pdf]
